# Aldolase A Enhances Intrahepatic Cholangiocarcinoma Proliferation and Invasion through Promoting Glycolysis

**DOI:** 10.7150/ijbs.59068

**Published:** 2021-04-23

**Authors:** Xiang Li, Chang Yu, Yichun Luo, Jiacheng Lin, Fang Wang, Xuehua Sun, Yueqiu Gao, Weifeng Tan, Qiang Xia, Xiaoni Kong

**Affiliations:** 1 Department of Hepatic Surgery, Renji Hospital, School of Medicine, Shanghai Jiaotong University, Shanghai, China.; 2 Central Laboratory, Department of Liver Diseases, ShuGuang Hospital Affiliated to Shanghai University of Chinese Traditional Medicine, Shanghai, China.

**Keywords:** Intrahepatic Cholangiocarcinoma, Aldolase A, Metabolism Reprogram, Enzymatic Activity, Glycolysis.

## Abstract

Energy metabolism reprogramming has been implicated in tumorigenesis and development. Key metabolism enzyme Aldolase A (ALDOA) has been shown to be highly expressed and involved in various kinds of cancers including hepatocellular carcinoma. In this study, we found that ALDOA was highly expressed in clinical intrahepatic cholangiocarcinoma (ICC) tissues, and its high expression was negatively correlated with overall survival (OS) and recurrence-free survival (RFS) in ICC patients. Knockdown of ALDOA expression significantly inhibited the proliferation and migration of ICC both *in vitro* and *in vivo*, while highly-expressed ALDOA in ICC cells promoted the proliferation and migration of ICC cells. By applying ALDOA inhibitor and metabolic mass spectrometry tests, we demonstrated that ALDOA modulated the biological characteristics and metabolic level of ICC cells depending on its enzymatic activity. In summary, ALDOA promotes ICC proliferation and migration by enhancing ICC cells glycolysis. Blocking enzymatic activity of ALDOA provides a strategy to inhibit ICC.

## Introduction

Primary liver cancer is one of the most common malignancies of the digestive system. According to its pathological type, primary liver cancer can be divided into hepatocellular carcinoma (HCC) and intrahepatic cholangiocarcinoma (ICC) 1. ICC is a kind of malignant tumor that arises from the epithelial cells of segmental or proximal branches of the bile duct. The incidence rate of ICC accounts for 15% of all primary liver malignancies, and it is the second most common liver cancer after HCC 2. Although the incidence of ICC is less than HCC, the degree of malignancy is higher, the treatment tolerance is higher, and the prognosis is poorer 3. Therefore, ICC is a difficult clinical problem that needs to be solved. There are many studies on the pathogenesis of intrahepatic cholangiocarcinoma, but no clear pathogenesis or effective treatment has been proposed.

Having an abnormal metabolic level is one of the main characteristics of tumor cells 4, 5. As early as in the 1920s, Otto Warburg proposed that malignant tumors mainly generate energy through glycolysis even in the presence of oxygen recombination rather than tricarboxylic acid cycling or oxidative phosphorylation. This phenomenon is also known as the Warburg effect 6. Currently, metabolic abnormality, chiefly the Warburg effect, is thought to play an important role in oncogenesis and tumor progression in a variety of tumors 7. Many glycolytic enzymes such as PKM2 and HK have been proved to influence tumor progression by participating in the regulation of tumor signal transduction 8, acting as a phospho-tyrosine-binding protein 9, or influencing protein-protein interaction (PPI) of tumor cells as a coactivator 10.

Aldolase A (ALDOA) is a member of the aldolase family, mainly involved in the glycolysis pathway, catalyzing the reversible conversion of β-D-fructose-1,6-bisphosphate to D-glyceraldehyde 3-phosphate and dihydroxyacetone phosphate 11. Although the function of ALDOA in tumor progression has been intensively studied, the role and mechanism of ALDOA in liver cancer, especially in ICC, has not been studied yet. Through statistical analysis of clinical TCGA and GEO databases, we found that in liver tumors (including HCC and ICC samples), the expression level of ALDOA was significantly higher than that of adjacent cancer or normal liver, and the expression of ALDOA was significantly and negatively related to the poor prognosis of patients (Supplementary Figure 1), suggesting that ALDOA might play an important function in liver cancer.

In this study, we validated the expression levels of ALDOA in clinical ICC samples and revealed a significant correlation between ALDOA expression and the survival rates of patients. Knockdown of ALDOA significantly inhibited the proliferation and migration of ICC cells, while exogenously-induced overexpression of ALDOA enhanced the proliferation of tumor cells. By using an inhibitor (Itaconate) to repress the enzymatic activity of ALDOA, we found that the tumor proliferation and invasion were suppressed, suggesting that ALDOA promotes ICC cell proliferation through its enzymatic activity. Further, by testing the glycolytic function with seahorse and detecting downstream metabolites of the glycolysis pathway by mass spectrometry, we found that the knockdown of ALDOA expression in tumor cells leads to a lower level of glycolytic products compared to that of control cells. All these findings may help to better understand the pathogenesis of ICC and provide a new target for ICC treatment.

## Materials and methods

### Clinical specimens and cell lines

The human tumor and paired para-tumor tissues and ICC specimens of the validation cohort were obtained from ICC patients undergoing hepatectomy at Renji Hospital affiliated to Shanghai Jiaotong University School of Medicine. Patient samples were obtained following informed consent and protocols that were approved by the ethical review committee of the World Health Organization's Collaborating Center for Research in Human Production (authorized by Shanghai Municipal Government). The cholangiocarcinoma cell line RBE, HuCCT1 and FRH-0201 were purchased from the Cell Bank of the Chinese Academy of Sciences (Shanghai, China).

### Cell culture

The ICC cell lines were cultured in RPMI 1640 medium (Gibco, C11875500CP) plus 10% (v/v) FBS (Gibco, 10099141C) plus Penicillin 100 U/mL and Streptomycin 100μg/mL (Gibco, 15140122) at 37°C and 5% CO_2_.

### RNA extraction, cDNA synthesis and qPCR

Total RNA was extracted by total RNA rapid extraction kit (Bioteke Corporation, RP4002). A total of 500 ng RNA was reverse-transcribed using HiScript II Q RT SuperMix for the qPCR kit (Vazyme, R222-01). The cDNA was diluted (1:10) for qPCR using ChamQ SYBR qPCR Master Mix (Vazyme, Q311-02) with gene-specific primers. Results were normalized by β-actin control. The primer sequences are as follows: ALDOA-Forward: 5'-GCTGTCACTGGGATCACCTTC-3', ALDOA-Reverse: 5'-GCTCGGAGTGTACTTTCCTTGA-3'; β-actin Forward: 5'-ctccatcctggcctcgctgt-3', β-actin Reverse: 5'-gctgtcaccttcaccgttcc-3'.

### Protein extraction and Western blot

Cells or tumor tissues were lysed on ice with RIPA (Thermo Scientific, 89900) containing protease inhibitor (MCE, HY-K0010) for 30 mins. After centrifugation for 10 mins at 12000 rpm, the supernatants were transferred to new EP tubes. The protein samples were quantified by the BCA method. Then samples were denatured at 100 ℃ for 10 mins. Protein extracts were resolved on 12% SDS-PAGE gels, transferred to nitrocellulose membranes (0.45 mm) after electrophoresis, and immunoblotted with special primary antibodies. Images were developed with ECL (GE Health Care, USA). The ALDOA antibody (11217-1-AP) was purchased from Proteintech. The β-actin antibody with HRP (A3854) and the anti-flag M2 antibody (F1804) were purchased from Sigma.

### Tissue microarrays and immunohistochemical staining

The ICC tissue microarrays contain 52 matched pairs of specimens. Strict pathological diagnoses and post-operative follow-ups were performed for all patients. IHC staining was performed to detect the expression of ALDOA. Sections were scored as positive if tumor cells showed a staining reaction in the cytoplasm and/or the nucleus. A quantitative score was given by estimating the percentages of positive cells: 0, (0-5%); 1, (5%-25%); 2, (25%-50%); 3, (50%-75%); and 4, (75%-100%). The intensity of positive staining was given scores as negative (0), pallide-flavens grains (1), buffy grains (2), and brown-black grains (3) respectively. Finally, total scores (0-7) for each sample were determined by the combination of the quantitative scores time the intensity scores. Score 0-4 were classified as low and score 5-7 were classified as high ALDOA expression respectively. Prior patient consent and approval from the Institutional Research Ethics Committee were obtained.

### RNA interference experiments

The siRNAs targeting homo ALDOA as well as negative control were designed and synthesized by GenePharma company. The siRNA oligos of homo ALDOA are shown as below: siRNA-1: GUGUCAUCCUCUUCCAUGATT, UCAUGGAAGAGGAUGACACTT; siRNA-2: GGCGUUGUGUGCUGAAGAUTT, AUCUUCAGCACACAACGCCTT. ICC cells were transfected in 6-well plates with Lipofectamine RNAiMAX (Thermo Fisher, 13778150) according to the manufacturer's protocol. Cells were collected for RNA and protein level verification and cell functional tests.

### Construction of stable transfer cell lines

The lentiviral vector was used to construct stable low expression transfer cell lines of ALDOA. After verifying the knock inefficiency of the siRNAs, the oligos of siRNA-1 and siRNA-2 were used to clone into the shRNA. Scramble shRNA was used as a negative control. ICC cell line RBE was infected with lentivirus [multiplicity of infection (MOI) = 20]. Stable cell lines were obtained by puromycin (2ug/ml) screening after lentivirus infection.

### Construction of high expression cell lines

Transcript NM_001127617.2 of ALDOA was used to construct ALDOA overexpression plasmid, and pcDNA3.1(+) vector was used as the negative control. ICC cell lines FRH-0201 and HuCCT1 were transfected with pcDNA3.1-ALDOA or pcDNA3.1-negative control vector in 6-well plates with Lipofectamine 3000 (Thermo Fisher, L3000015) according to the manufacturer's protocol. Cells were collected for RNA and protein level verification and cell functional tests.

### CCK-8 proliferation assay

Cell proliferation was assayed by the Cell Counting Kit-8 assay (Dojindo, CK04). Briefly, 100 uL cells suspension (1 × 10^4^ cells/ml) were plated in 96-well plates overnight for attachment. 10 uL CCK-8 reagents were added and cells were incubated at 37°C for 2 hrs before being measured at the wavelength of 450 nm by the microplate reader. All experiments were performed independently at least 3 times.

### Wound healing assay

Cells were cultured in 6-well plates. When the wells were fully confluent, a linear wound was scraped by a pipette tip in each well. Cells were washed twice to remove detached cells and debris. Wound size was observed and measured under brightfield microscopy. All experiments were performed independently at least 3 times.

### Invasion assay

ICC cells were seeded into 24-well Transwell chambers coated with Matrigel (1/8 diluted, BD Biosciences, 354234). The invasion of the cells through the Matrigel to the underside of the chambers was assessed 24 h. Cells were fixed by 4% paraformaldehyde for 30 mins and then stained with 0.1% crystal violet for 15 mins. Cells of the inner chamber were wiped with cotton swabs. Pictures of the cells were observed and took under brightfield microscopy.

### Colony formation assay

After digestion and count, 1000 cells were seeded and incubated in a fresh 6-well plate for 14 days for the formation of the colonies. Colonies were fixed by 4% paraformaldehyde for 30 mins and then stained with 0.1% crystal violet for 15 mins.

### Xenografting nude mice model

Four to six weeks old male nude mice were purchased from Shanghai SLAC Laboratory Animal Co., Ltd for tumor cell xenograft assay. Nude mice were randomly assigned to 3 groups and 5 × 10^6^ cells were resuspended in 50 μL phosphate-buffered saline (PBS) and subcutaneously injected into the left armpit of these nude mice. Tumors were measured twice a week and calculated by the following formula: Volume = 0.5 × Length × Width^2^. Six weeks after tumor cell injection, mice were sacrificed. The xenografted tumors were thoroughly examined. All procedures involving animals were approved and performed in accordance with the Animal Care and Use Committee of Shanghai Jiaotong University.

### Glycolysis stress analysis

Cellular glycolytic capacity was measured using the Seahorse Bioscience XFe96 Extracellular Flux Analyzer (Seahorse Bioscience, North Billerica, MA), according to the manufacturer's instructions of seahorse XFe Cell Glycolysis Stress Test Kit. Briefly, 1 × 10^4^ RBE cells and HuCCT1 cells were seeded in Seahorse XFe96 well culture plates (Bucher Biotech AG, Basel, Switzerland) and incubated at 37℃/5% CO_2_ overnight. Cells were washed with Seahorse XF Glycolysis Stress Test Assay Medium (1640 without phenol red containing 2 mM glutamine) twice and were placed in a 37℃ non-CO_2_ incubator for 1 hour prior to the assay. 20 μL of 100 mM Glucose, 22 μL of 20 μM Oligomycin, and 25 μL of 500 mM 2-deoxy-glucose (2-DG) were automatically injected to measure ECAR. ECAR values were calculated after normalizing with the cell number and plotted as the means ± SD.

### Metabolites spectrum detection

Cells were removed from the culture medium and washed with precooled PBS 2-3 times. After discarding the supernatant, 1 mL 60% precooled chromatogram methanol was added. Cells were scraped and centrifuged at 1000 g at 4℃ for 1min. The supernatant was discarded, and cell precipitation was collected. Cells were quick-frozen by liquid nitrogen for 15mins and then detected by the mass spectrometer.

### Statistical analysis

All statistical analyses were performed using Graphpad Prism 8 and SPSS 17.0 software. Results were expressed as means ± standard deviation. The Student's t-test was used to compare the difference between the two groups. The Pearson χ^2^ test or the Student's t-test was used for analyzing the relationship between the ALDOA expression and the clinicopathologic features. Survival analysis was determined using the Kaplan-Meier analysis and Cox regression. P values less than 0.05*, less than 0.01**, or less than 0.001*** were considered statistically significant.

## Results

### The expression level of ALDOA in ICC samples is increased and is significantly associated with poor prognosis

**Figure 1:**
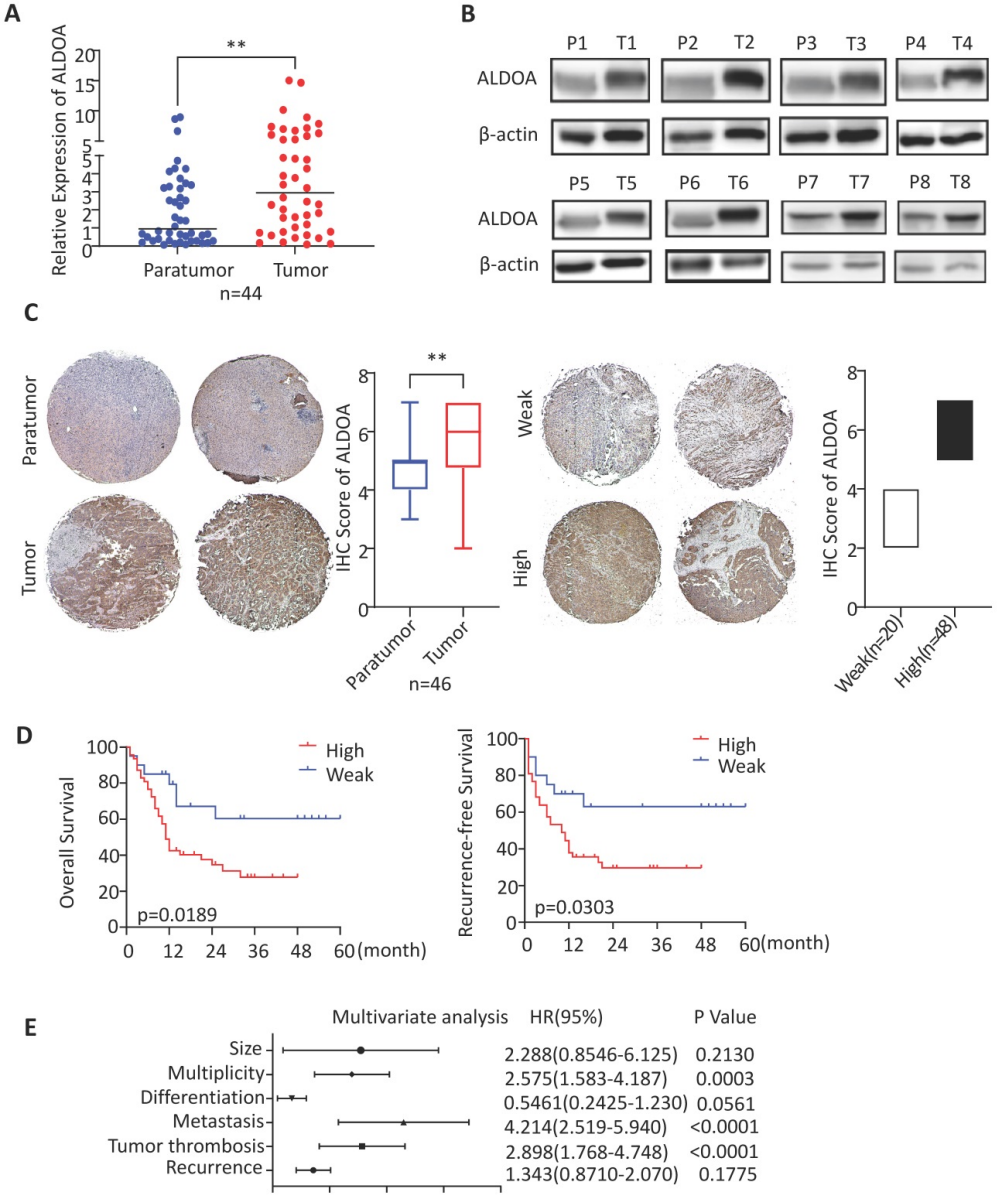
** ALDOA is highly expressed in ICC and is significantly associated with patient prognosis.** A. mRNA expression level of ALDOA in tumor and adjacent paratumor tissues. B. Detection of protein expression level of ALDOA in tumor (T) and adjacent paratumor (P) tissues by Western blot. C. Representative Immunohistochemistry images of ALDOA staining scores in tumor and paratumor tissue samples (× 40 magnification). D. Kaplan-Meier survival curve of patients with different ALDOA expression levels. E. Forest plot based on the results of multivariate analysis of the factors associated with overall survival of ICC patients. Abbreviation: HR, hazard ratio. Results are shown as means ± SD. (**, P < 0.01).

ALDOA has been found to have elevated expression levels in a variety of solid tumors. To validate the expression level of ALDOA in ICC tissues, we collected clinical tumor tissue samples from patients, who were treated surgically and diagnosed post-operatively with ICC at Renji Hospital. By measuring the RNA and protein levels in these tissues, we found that, in ICC tissue samples, ALDOA was significantly overexpressed (Figure 1A-B). The tissue microarray immunohistochemical detection and analysis further verified the high expression of ALDOA in ICC tissues (Figure 1C, Supplementary Figure 1D), and found that the increased expression of ALDOA was significantly correlated with the malignant degree of the tumor and the poor prognosis of the patients (Figure 1D). In Figure 1D, all cases included in this trial were divided into the high ALDOA expression group and low ALDOA expression group according to the IHC staining score criteria in Figure 1C. The statistical analysis revealed that there were significant differences in overall survival and recurrence-free survival rates between the two groups (P = 0.0189, P = 0.0303). As can be seen from the Kaplan-Meier curves, the overall survival rate of the patients with high ALDOA expression began to decline at about 6 months after surgery, and the survival rate was significantly lower than that of the patients with low ALDOA expression after 48 months. During 48-60 months after surgery, statistics showed that most of the high ALDOA expression patients had a poor prognosis. Thus, high ALDOA expression ICC patients had a significantly poor prognosis. Multivariate regression analysis of clinical prognostic information demonstrated that ALDOA expression level may be correlated with multiplicity and tumor metastasis (Figure 1E). The above results are consistent with the expression level of ALDOA and patient prognosis in the liver tumor database (Supplementary Figure 1A-C).

### Knockdown of ALDOA inhibits the proliferation, migration and invasion of tumor cells *in vitro*

**Figure 2:**
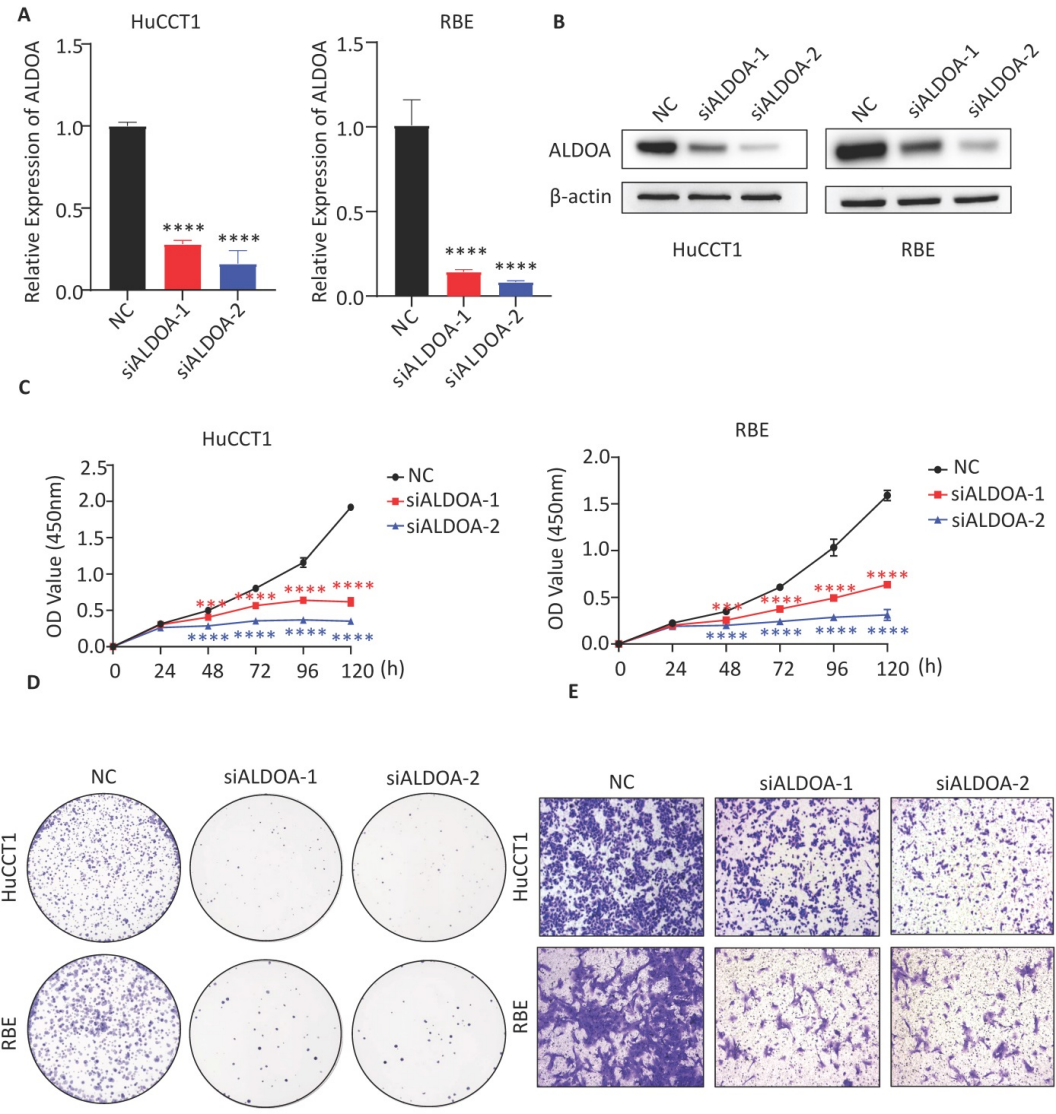
** The biological function of tumor cells was significantly inhibited after ALDOA knockdown.** A. ALDOA mRNA levels of tumor cells after ALDOA were knocked down by siRNA. B. ALDOA protein levels of tumor cells after ALDOA were knocked down by siRNA. C. CCK-8 assay was used to detect the proliferation level of tumor cells after siRNA knockdown ALDOA expression. D. Clonal formation was used to detect the proliferation level of tumor cells after siRNA knockdown ALDOA expression. E. Transwell assay detected the invasion level of tumor cells after siRNA knockdown of ALDOA expression. Results are shown as means ± SD. (***, P < 0.001; ****, P < 0.0001).

To verify the effect of ALDOA expression level on the biological function of tumor cell lines *in vitro*, the expression level of ALDOA in each ICC cell line was determined. Western blot assay showed a relatively high level of ALDOA expression in RBE and HuCCT1 and a relatively low level of ALDOA expression in FRH-0201 (Supplementary Figure 2A). Therefore, RBE and HuCCT1 cell lines were chosen for verifying the changes in the cell function after ALDOA knockdown. Two siRNA were used to knock down the expression level of ALDOA and the knockdown efficiency of siRNA was verified by qPCR and Western blot (Figure 2A-B). CCK-8 test illustrated that the knockdown of ALDOA significantly inhibited the proliferation of tumor cell lines (Figure 2C). The Colony formation assay also confirmed that the proliferation and colony formation ability of RBE and HuCCT1 cell lines were decreased significantly after ALDOA knockdown (Figure 2D). Furthermore, the Transwell assay was used to detect the migration and invasion capabilities of tumor cells. The results showed that the migration and invasion capabilities of RBE and HuCCT1 were significantly impaired after ALDOA knockdown (Figure 2E). Further Wound Healing assay showed that RBE and HuCCT1 migration were inhibited after ALDOA knockdown (Supplementary Figure 2B).

### Knockdown of ALDOA expression inhibits tumor cell formation *in vivo*

**Figure 3:**
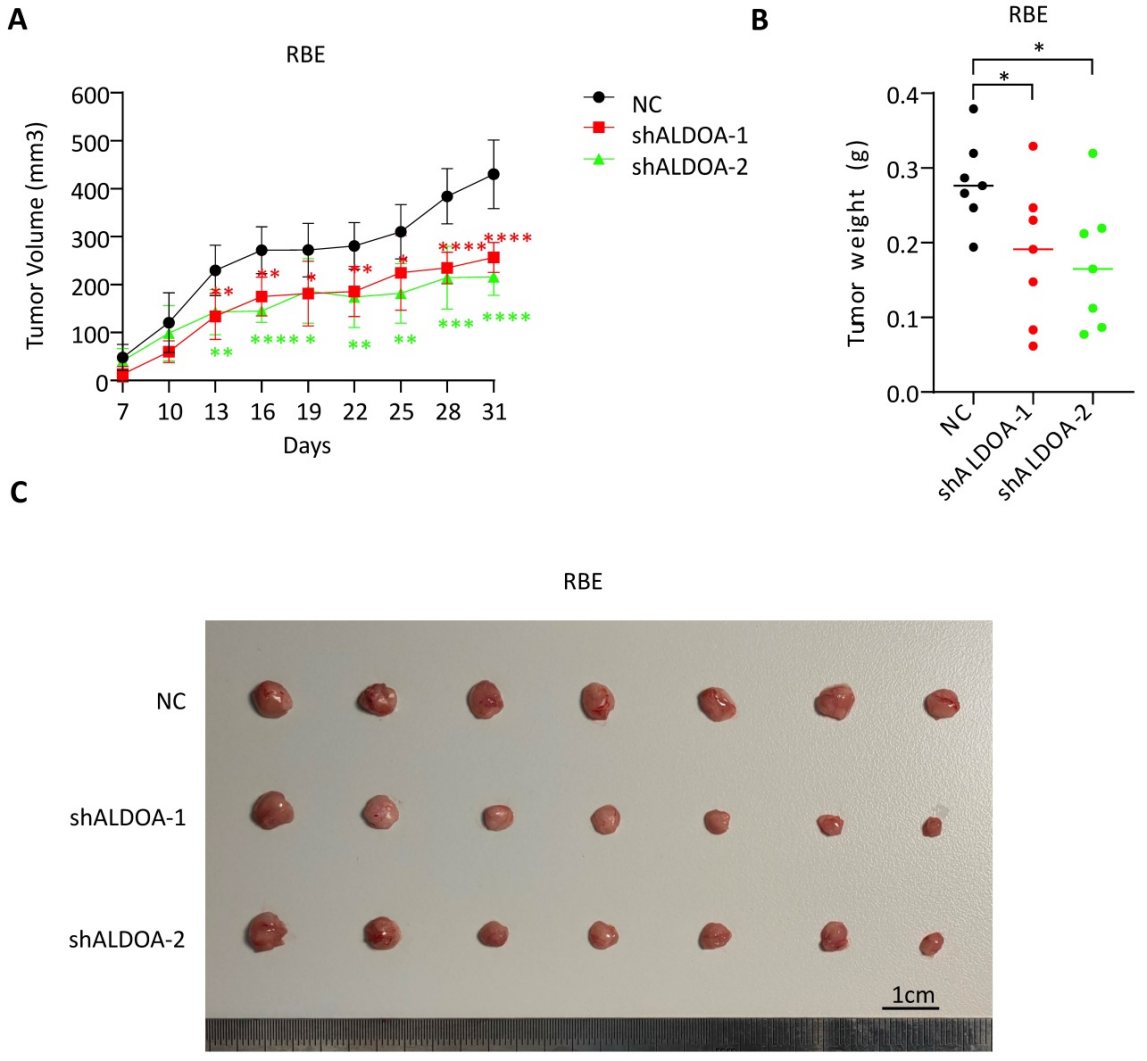
** Subcutaneous tumor formation was significantly inhibited after ALDOA knockdown.** A. Tumor growth curves of RBE with stable ALDOA knockdown in subcutaneous tumor formation mouse model. B. Tumor weights of subcutaneous tumor formation after mice sacrificed. C. Photography of the tumors in subcutaneous model mice. Scale bar = 1cm. Results are shown as means ± SD. (*, P < 0.05; **, P < 0.01; ***, P < 0.001; ****, P < 0.0001).

Since the knockdown of ALDOA significantly inhibited proliferation and invasion of ICC tumor cell lines *in vitro*, we further examine the role of ALDOA in tumor cell proliferation and tumorigenesis *in vivo*. A stable ALDOA knockdown RBE tumor cell line was constructed with lentiviruses and a nude mouse subcutaneous xenograft model was established with this ALDOA-konckdown RBE cell line. The results showed that tumor growth rate and tumor size were significantly inhibited after ALDOA knockdown compared to the negative control group (Figure 3A-C). This result is consistent with the results of *in vitro* experiments.

### Overexpression of ALDOA can promote proliferation and invasion of tumor cells

**Figure 4:**
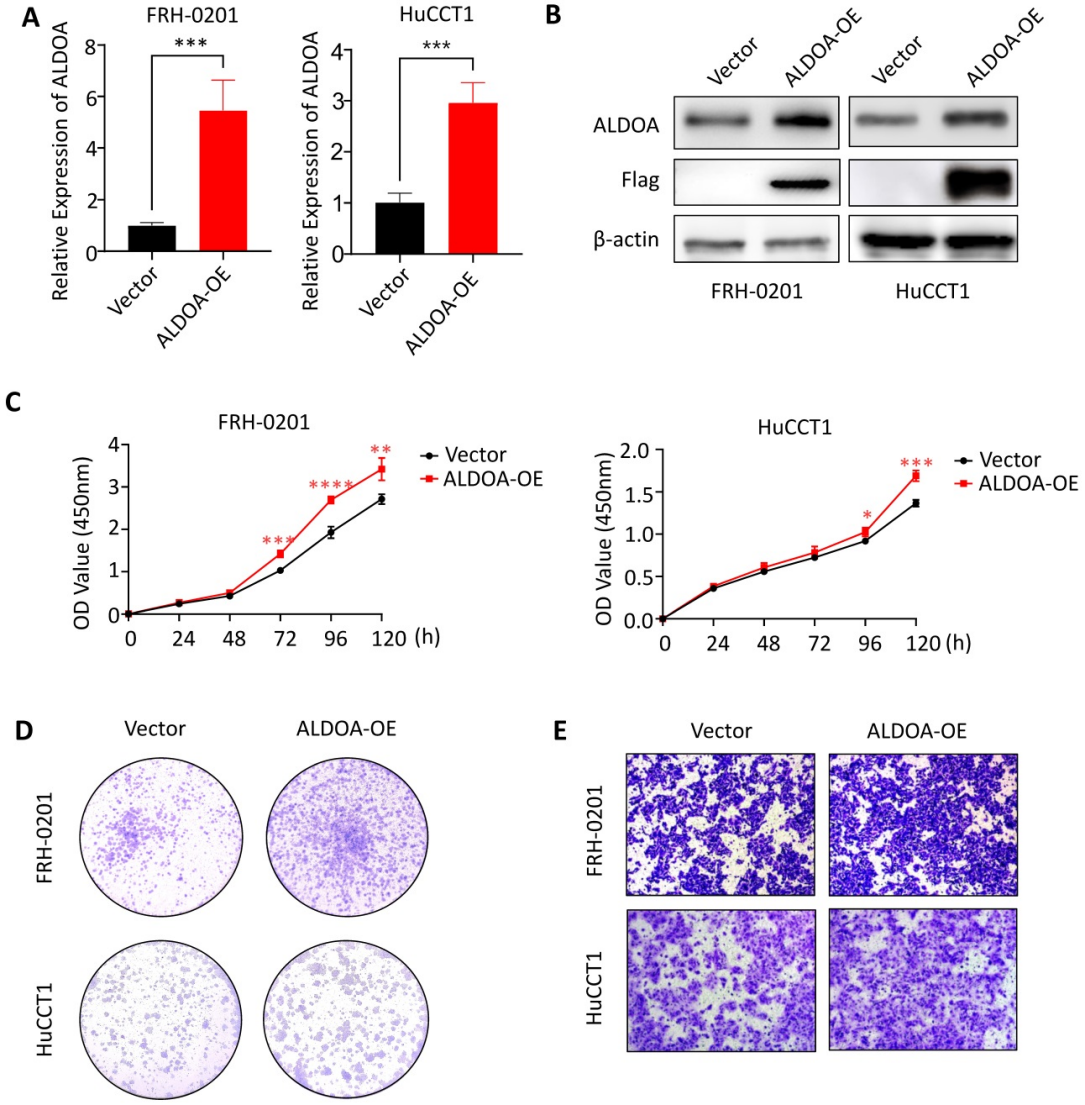
** The proliferation and invasion of tumor cells were enhanced after overexpression of ALDOA.** A. ALDOA mRNA levels of tumor cells after ALDOA was over expressed by plasmid. B. ALDOA protein levels of tumor cells after ALDOA were over expressed by plasmid. C. CCK-8 assay was used to detect the proliferation level of tumor cells after over expressed of ALDOA by plasmid. D. Clonal formation was used to detect the proliferation level of tumor cells after over expressed of ALDOA by plasmid. E. Transwell assay detected the invasion level of tumor cells after over expressed of ALDOA by plasmid. Results are shown as means ± SD. (*, P < 0.05; **, P < 0.01; ***, P < 0.001; ****, P < 0.0001).

To further confirm the relationship between ALDOA expression and tumor progression, we overexpressed ALDOA in low-ALDOA-expressing FRH-0201 cell line and high-ALDOA-expressing HuCCT1 cell line by plasmid respectively. The qPCR and Western blot were used to verify the overexpression efficiency (Figure 4A-B). CCK-8 test showed that the proliferation rate of ALDOA-overexpressing FRH-0201 and HuCCT1 cells increased to some extent (Figure 4C). The Colony formation assay also showed that both modified cell lines had stronger proliferation and colony formation ability, especially the ALDOA-overexpressing FRH-0201 cells with a originally lower ALDOA expression level (Figure 4D). In terms of migration and invasion ability, the Transwell assay and Wound Healing assay showed that the migration and invasion ability of ALDOA-overexpressing cells were also improved to some extent (Figure 4E, Supplementary Figure 3). In conclusion, it can be seen that the proliferation, invasion and migration ability of tumor cells are improved to different degrees after ALDOA overexpression, but the level of improvement is not very significant. This may be because that tumor cells themselves have a strong ability to proliferate and invade, and the pathway that ALDOA is involved in cannot completely determine the progress of the tumor. Therefore, partially increasing the expression level of ALDOA has limited influence on the progression of the tumor.

### Proliferation and invasion ability of ICC cells were inhibited after inhibiting the enzyme activity of ALDOA by Itaconate

**Figure 5:**
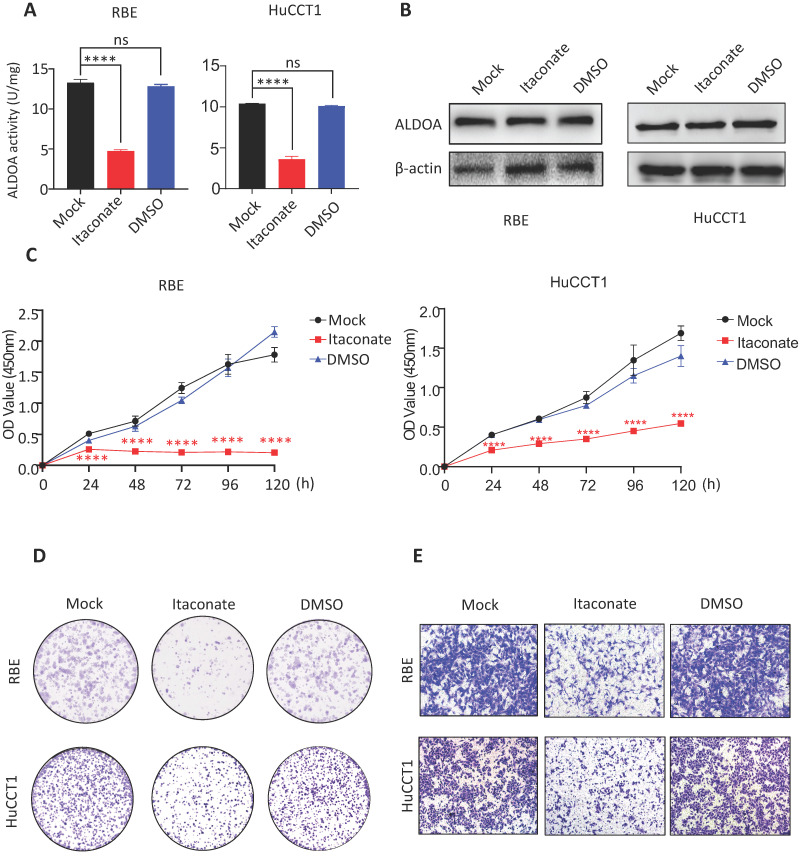
** The proliferation and invasion of tumor cells declined after inhibiting enzyme activity of ALDOA by Itaconate.** A. Enzyme activity of ALDOA of tumor cells after treated by Itaconate or DMSO for 24h. B. ALDOA protein levels of tumor cells after treated by Itaconate or DMSO for 24h. C. CCK-8 assay was used to detect the proliferation level of tumor cells after treated by Itaconate or DMSO for 24h. D. Clonal formation was used to detect the proliferation level of tumor cells after treated by Itaconate or DMSO for 24h. E. Transwell assay detected the invasion level of tumor cells after treated by Itaconate or DMSO for 24h. Results are shown as means ± SD. (ns, no significance; ****, P < 0.0001).

ALDOA is not only involved in cell signal transduction but also an important rate-limiting enzyme in the glycolytic pathway. In order to further reveal the mechanism behind the tumor-inducing function of ALDOA overexpression, we hope to inhibit the catalytic activity of ALDOA in the glycolysis pathway through its specific enzyme activity inhibitor and try to observe its effect on tumor cell proliferation and invasion. According to Qin's previous studies, Itaconate can inhibit ALDOA's enzymatic activity by modifying Cys73 and Cys339 of the ALDOA without changing its protein expression level^12^. Therefore, we used Itaconate to inhibit the enzymatic activity of ALDOA (Figure 5A) without changing the protein expression level of ALDOA (Figure 5B), and tested the proliferation and invasion abilities of ICC cells by methods such as CCK-8 and Transwell (Figure 5C-E, Supplementary Figure 4). The results proved that, without changing the expression level of ALDOA protein, inhibiting its enzyme activity alone could affect the ICC cells' abilities to proliferate and invade. Furthermore, they also illustrated that, during the progression of ICC, ALDOA mainly relies on its enzyme activity to exert its regulatory effect on tumor progression.

### ALDOA mainly affects the biological function of tumor cells by restricting the glycolysis pathway

**Figure 6:**
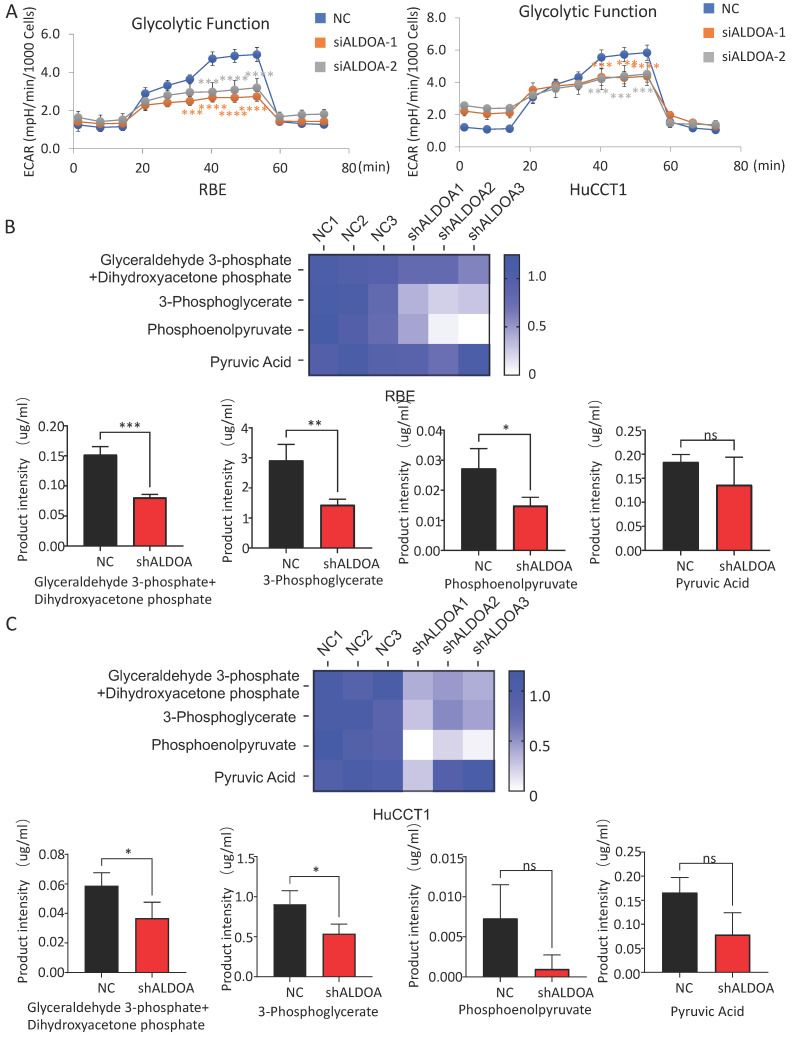
** Effect of ALDOA expression level on the metabolic function of tumor cells.** A. Seahorse was used to detect the glycolysis level of tumor cells with siRNA knockdown of ALDOA expression. B. The changes of metabolites in the glycolytic pathway after ALDOA knockdown were detected by mass spectrometry in RBE cell line. C. The changes of metabolites in the glycolytic pathway after ALDOA knockdown were detected by mass spectrometry in HuCCT1 cell line. Results are shown as means ± SD. (ns, no significance; *, P < 0.05; **, P < 0.01; ***, P < 0.001; ****, P < 0.0001).

The above results showed that the high expression of ALDOA in ICC cells mainly plays a regulatory role through its enzymatic activity. However, whether the regulation by ALDOA can affect the level of glycolysis in tumors still needs further verification. In order to detect whether changes in ALDOA expression levels actually affect glycolysis levels, we detected the level of glycolysis metabolism in cells by Seahorse Glycolysis Stress test and Cell Mito Stress test. There are many methods to measure glycolysis levels, such as Glycolysis Assay Kit from ABCAM and Glycolysis metabolism assay kits from Merck. Among them, Agilent's Seahorse Glycolysis Stress Test Kit is considered to be the most direct and accurate method for the detection of glycolysis level, and it has been widely used in relevant researches 13, 14. Mitochondrial respiration and glycolysis are two major pathways of cellular energy metabolism. Seahorse measures cellular respiration, glycolysis, and ATP production by detecting changes in O_2_ consumption rate (OCR) and extracellular acidification rate (ECAR) in analytes using unlabeled sensors. Seahorse is one of the most effective ways to detect the metabolism of living cells in real time. Seahorse Glycolysis Stress test showed that ALDOA knockdown significantly inhibited the glycolysis level of tumor cells (Figure 6A) but barely affected the mitochondrial metabolism according to the Cell Mito Stress test (Supplementary Figure 5B-C).

The glycolytic pathway is a series of enzyme-catalyzed reactions. As one of the catalytic enzymes, ALDOA is mainly involved in catalyzing 1, 6-diphosphate fructose to produce glyceraldehyde 3-phosphate and dihydroxyacetone phosphate, which are intermediate products of glycolysis pathway (Supplementary Figure 5A). Whether the change in ALDOA expression level will have an impact on the generation of downstream products still needs further verification. Mass spectrometry has a wide dynamic range, can perform reproducible quantitative analysis, and can analyze complex physiological body fluids. At present, it is widely used in metabolomics researches 15, 16. We detected key downstream metabolites in the glycolytic pathway of tumor cells by metabolic mass spectrometry. The results showed that not only the content of glyceraldehyde 3-phosphate and dihydroxyacetone phosphate, the direct metabolites of ALDOA, but also the intermediate metabolites in the downstream glycolysis pathway decreased significantly in both RBE and HuCCT1 (Figure 6B-C). However, it can be also found that the levels of some detected downstream products have decreased, but due to the limitation in sample size and large fluctuations within the group, the difference in value cannot be calculated. This part of the result needs to be further investigated.

## Discussion

ICC is one of the hepatic tumors that have a high malignant degree and poor prognosis. Due to the increase in morbidity and mortality rate over the past 20 years, ICC has gradually become the focus of research 17. Currently, there is no effective therapy for ICC except surgical treatment. However, even after curative-intent surgery, the recurrence rate is as high as 40%-80% 18, the 5-year overall survival (OS) rarely exceeded 30% to 35%, and the median aggregate overall survival was only approximately 28 months^19^. This is not only due to its high degree of malignancy but also because of the difficulties in early diagnosis, rapid progression of the disease and high tolerance to chemotherapy drugs. Therefore, there is an urgent need to better understand the underlying pathogenesis of ICC.

Although tumor metabolic abnormalities have long been discovered, they have not been the main research object of cancer biology. In the past 15 years, tumor metabolism, especially glucose metabolism, has gradually become a hot spot in cancer research 20. Currently, tumor metabolic reprogramming is considered to be one of the key influencing factors for tumor formation and development. Compared with normal cells, glucose metabolism is preferentially expressed as glycolysis in cancer cells, even in an oxygen-rich environment. This 'aerobic glycolysis' phenomenon was first described by Otto Warburg in the 1920s 21. The Warburg effect, which represents a shift in the use of glucose by tumor cells from oxidative phosphorylation to glycolysis, is considered to be a tumor-specific metabolic mode and has been verified in the carcinoma cells of various cancers, including lung cancer 22, breast cancer 23, colon cancer 24, renal carcinoma 25 and glioblastoma 26. This change in energy metabolism is regulated by complex factors, including pressure on the tumor microenvironment and genetic changes. The Warburg effect has become more and more attractive to researchers, and inhibiting the glycolysis of cancer cells has become an emerging cancer treatment strategy. However, the mechanism of the Warburg effect affecting tumorigenesis and progression has remained controversial. So far, there have been many studies on tumor progression and treatment targeting the glycolysis pathway. Unfortunately, in the researches about treating tumors by inhibiting the glycolysis pathway, glycolytic pathway inhibitors, such as 2-DG and Lonidamine, have not achieved the desired results 27, 28. The enhanced glycolysis of tumor cells is mainly due to the increased expression or activity of key glycolytic enzymes. In recent years, researchers are seeking to target tumors by inhibiting the activity of key enzymes in the tumor glycolysis pathway. Some studies have shown that inhibiting the tumor glycolysis pathway can effectively inhibit the proliferation of tumor cells and even play a role in killing tumor cells. Crucial enzymes in the glycolysis pathway, such as Hexokinase 2 (HK2) 29, 30, M2-type acetone kinase (PKM2) 8, 31 and phospho-fructosidase (PFK) 32, 33, have become tumor markers. Their expression and activity have been proved to be able to affect tumor cell glycolysis, which in turn affects tumor cell proliferation. Selective inhibitors for glycolytic enzymes, such as HK2, have been developed and are currently used in the treatment of lung cancer 34 and prostate cancer 35. As a selective inhibitor of PKM2, TT-232 (CAP-232) can promote the production of low-activity pyruvate kinase, leading to metabolic stress and death of cultured tumor cells, and is expected to be used in the treatment of various solid tumors 36. Therefore, we focused our attention on key enzymes in the glycolytic pathway and attempted to find new targets for inhibiting tumor proliferation and progression.

In the previous study, we analyzed GEO, TCGA and other databases for tumor metabolic genes and found that the expression level of glycolytic enzyme ALDOA in liver tumors, especially in ICC, was significantly increased. This abnormal expression level is significantly associated with the prognosis of tumor patients. Therefore, we verified its expression in clinical samples of liver tumors and found that the glycolytic enzyme ALDOA is highly expressed in ICC. Expression of ALDOA is related to the proliferation, invasion, metastasis and drug tolerance of ICC cells. We further proved that knockdown of the expression of ALDOA in RBE and HuCCT1 cells could reduce the cell's motility and tumorigenesis. These data indicate that ALDOA may be an important marker for the occurrence and development of ICC, and may become a therapeutic target for drug development.

ALDOA is a ubiquitous glycolytic enzyme that drives the glycolytic metabolism pathway in mammalian cells and is mainly expressed in adult muscle tissue. Overexpression of ALDOA has been observed in a variety of cancers including renal clear cell carcinoma 37, lung squamous cell carcinoma 38, colorectal cancer 39, osteosarcoma 40, and oral squamous cells 41, suggesting that glycolysis is enhanced in these cancer cells. However, the current expression of ALDOA in ICC is still unclear, and its regulatory mechanisms involved in tumor progression have yet to be studied. In the research of other solid tumors, some researchers pointed out that ALDOA mainly participates in the regulation of tumor development by involving in the regulation of cell signal transduction pathways, such as c-MYC/HIF-1 pathway 42, 43 or EMT pathway 44. Since ALDOA is a common metabolic enzyme, there is no research indicating whether it affects tumor progression in the form of enzymes. In our study, the proliferation and migration of tumor cells were significantly inhibited after inhibiting the catalytic activity of ALDOA enzyme by Itaconate without changing its expression level. By using Itaconate to inhibit the enzymatic activity of tumor cells ALDOA, we found that it has a significant impact on the proliferation and invasion of tumor cells, indicating that ALDOA is mainly involved in the progression of tumors through its catalytic activity, and its degree of activity plays a decisive role.

Itaconate was first identified as a metabolite of macrophage activation 45. Itaconate can negatively feedback regulate the inflammatory response by inhibiting the production of inflammatory factors that macrophages participate in. This process is mainly through the alkylation of KEAP1 to activate NRF2 46. Kong et al. found that 4-octyl itaconate (4-OI), which is the derivative of itaconate, can alkylate the sulfhydryl groups on proteins by directly acting on the 22 cysteine residues on GAPDH, inhibiting its enzymatic activity and reduces the release of inflammatory factors 47. And in the studies of some autoimmune diseases such as psoriasis, Itaconate were believed to play a role in the treatment of autoimmune diseases by inhibiting the systemic immune response 48. In tumor study, Itaconate was thought to indirectly regulate tumor progression through macrophages 49. It is not clear whether Itaconate can directly involve in tumor progression regulation. Until 2019, Qin et al. found that Itaconate can inhibit ALDOA's enzymatic activity by modifying Cys73 and Cys339 of ALDOA without changing its protein expression level 12. As an important metabolic enzyme in the glycolytic pathway, ALDOA has been shown to be involved in the regulation of the progression of a variety of tumors 37-41. Our study also confirmed that the high expression of ALDOA promoted the progress of ICC, and Itaconate can directly inhibit the glycolysis level of ICC tumor cells and thus affect tumor progression.

However, it is worth noticing that Itaconate has other regulatory targets besides ALDOA, and the possible regulatory mechanisms of Itaconate are complex. In addition to the commonly believed anti-inflammatory effect, according to the studies of Qin et al, Itaconate can not only inhibit the enzyme activity of ALDOA, but also modify two other key glycolytic enzymes (GAPDH and LDHA), which makes the mechanism of Itaconate participating in the process of tumor glycolysis complicated. Further studies are needed to investigate the role of the other two enzymes in tumor progression and confirm whether protein modifications of GAPDH and LHDA by Itaconate also play an important anti-tumor role. Alternatively, the study on specific enzymatic activity inhibitors of ALDOA can also change the limitations of current research.

## Conclusions

In conclusion, our data indicated that increased expression of glycolytic enzyme ALDOA plays an important role in the progression of ICC. The expression level of ALDOA was significantly correlated with the degree of malignancy and prognosis of ICC tumors. Knocking down the expression level of ALDOA or blocking its enzyme activity can significantly inhibit tumor progression. ALDOA plays a role in tumor mediation mainly through its enzyme activity level.

However, according to the results of this study, further in-depth research is needed. For example, the regulation mechanism of the upregulation of metabolic enzymes and the enzyme activity activation pathway of ALDOA are still unknown. Currently, there is no specific inhibitor targeting ALDOA enzyme activity. Furthermore, whether there are other enzymes in the glycolysis pathway that could affect tumor progression like ALDOA. These problems and their specific molecular mechanisms need further study.

## Supplementary Material

Supplementary figures.

## Abbreviations

ICC: Intrahepatic Cholangiocarcinoma
ALDOA: Aldolase A
OS: overall survival
RFS: recurrence-free survival
HCC: hepatocellular carcinoma
PPI: protein-protein interaction
siRNA: small interfering RNA
shRNA: short hairpin RNA
OCR: O2 consumption rate
ECAR: extracellular acidification rate
4-OI: 4-octyl itaconate
