# Supplementary material for: Aldolase A Enhances Intrahepatic Cholangiocarcinoma Proliferation and Invasion through Promoting Glycolysis
Source: Int J Biol Sci. 2021 Apr 23;17(7):1782–94. doi: 10.7150/ijbs.59068 (PMC8120471; doi:10.7150/ijbs.59068)
Supplement: Supplementary file 1 — Supplementary figures. [file ijbsv17p1782s1.pdf]

Supplementary materials

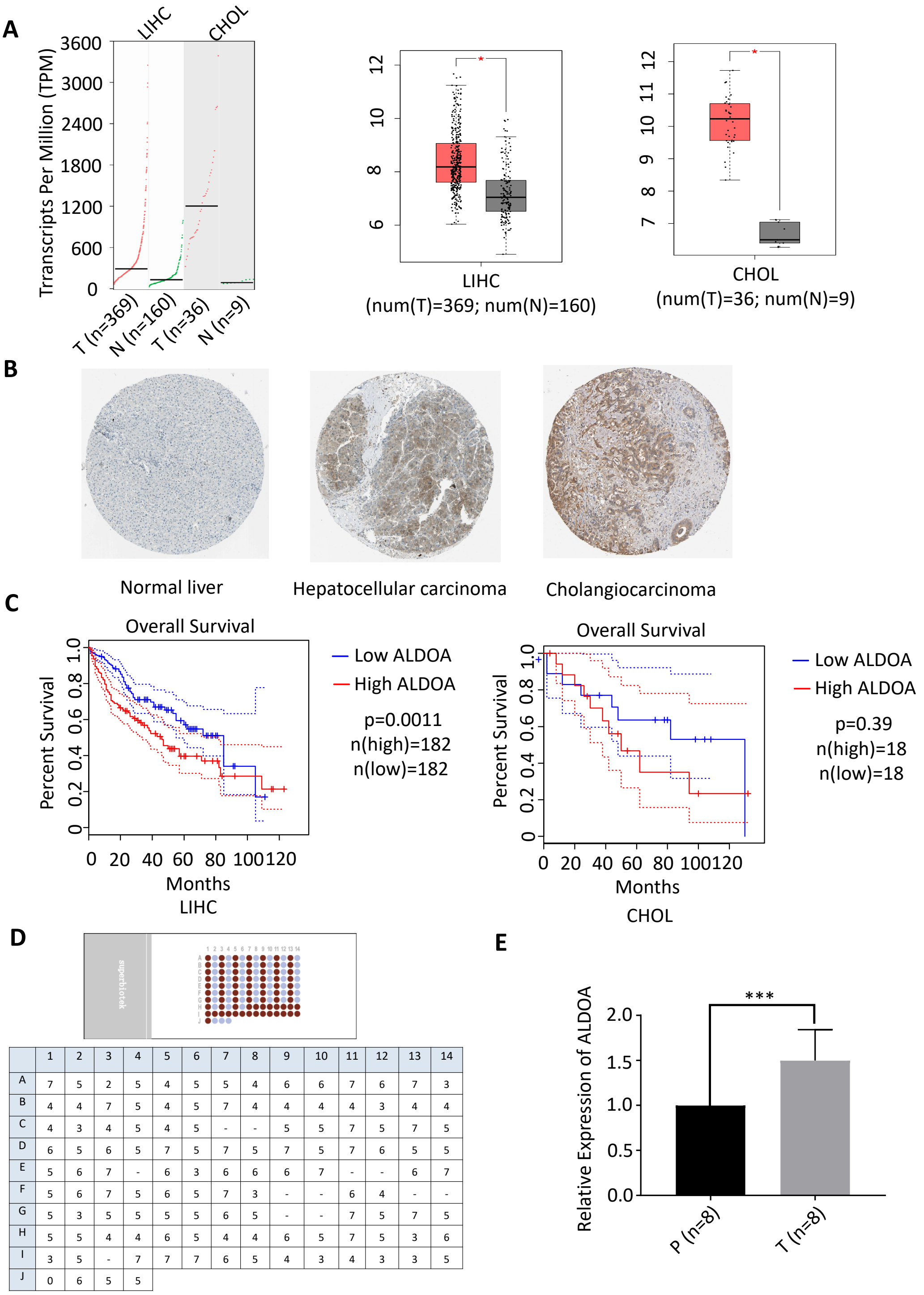

	1	2	3	4	5	6	7	8	9	10	11	12	13	14
A	7	5	2	5	4	5	5	4	6	6	7	6	7	3
B	4	4	7	5	4	5	7	4	4	4	4	3	4	4
C	4	3	4	5	4	5	-	-	5	5	7	5	7	5
D	6	5	6	5	7	5	7	5	7	5	7	6	5	5
E	5	6	7	-	6	3	6	6	6	7	-	-	6	7
F	5	6	7	5	6	5	7	3	-	-	6	4	-	-
G	5	3	5	5	5	5	6	5	-	-	7	5	7	5
H	5	5	4	4	6	5	4	4	6	5	7	5	3	6
I	3	5	-	7	7	7	6	5	4	3	4	3	3	5
J	0	6	5	5										

Supplementary Figure 1. The expression level of ALDOA in database. A. The mRNA expression level of ALDOA in tumor and adjacent tissues of hepatocellular carcinoma and cholangiocarcinoma (data was analysed by GEPIA according to TCGA and GEO). B. Protein expression level of ALDOA in normal liver tissue (patient id:1899), hepatocellular carcinoma tissue (patient id:2766) and cholangiocarcinoma tissue (patient id:937) by IHC (data from the human protein atlas). C. Survival curve of patients of hepatocellular carcinoma and

cholangiocarcinoma with different ALDOA expression levels（data was analysed by GEPIA according TCGA and GEO). D. The arrangement and immunohistochemical staining scores of tissue chip, red represents tumor tissue, and blue represents adjacent paratumor tissue (- indicates that IHC staining score cannot be accurately estimated due to factors such as tissue section damage). E. The Western blot results in Figure 1B were quantified by Image J and analysed by Graphpad. Results are shown as means ± SD. (*, P < 0.05; ***, P<0.001).

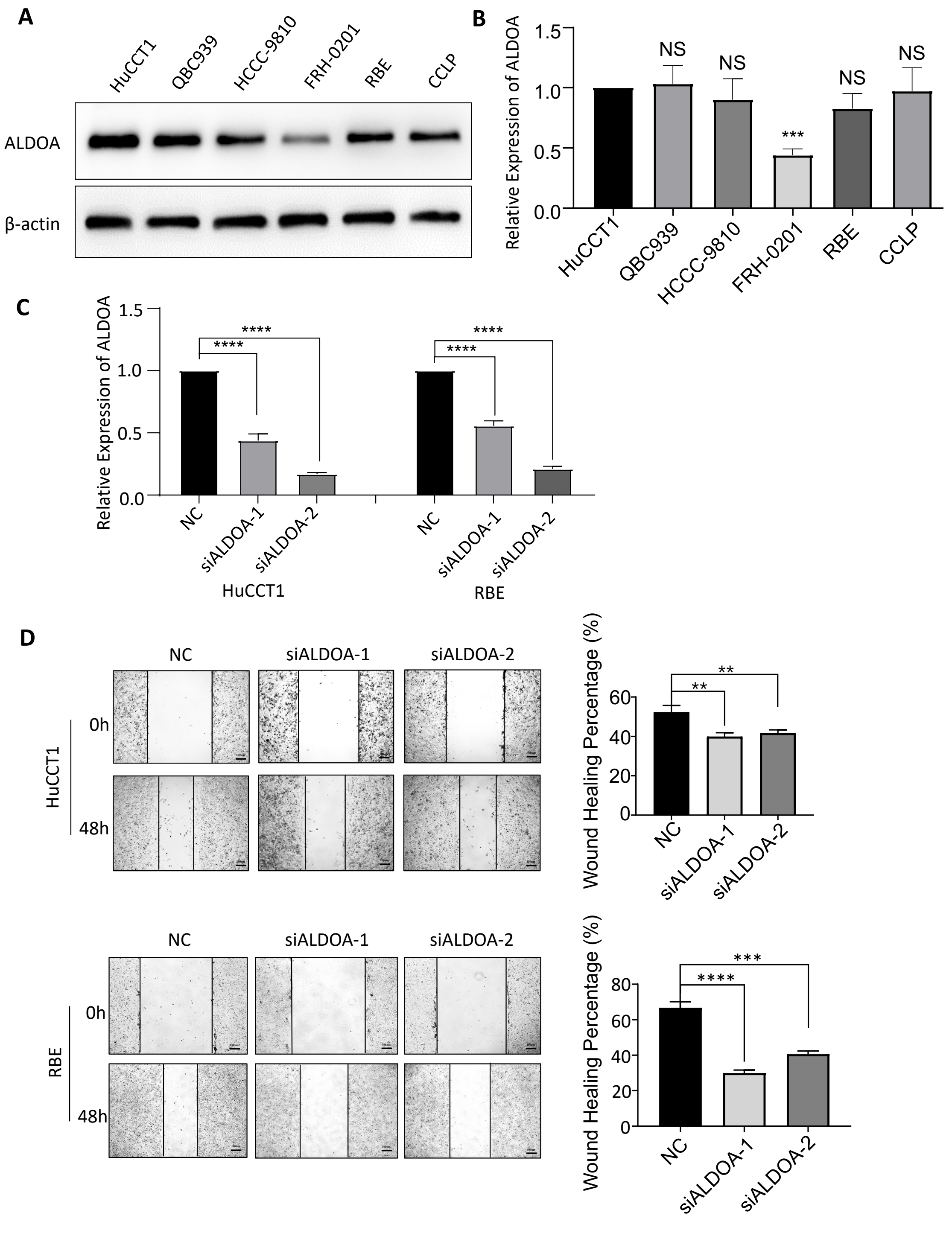

Supplementary Figure 2. A. ALDOA expression level in ICC cell lines. B. The Western blot results in Figure S2A were quantified by Image J and analysed by Graphpad. C. The Western blot results in Figure 2B were quantified by Image J and analysed by Graphpad. D. The level of tumor cell invasion after knockdown of ALDOA by siRNA detected by Wound healing test. Results are shown as means ± SD. (**, P<0.01; ***, P < 0.001; ****, P<0.0001). Scale bar, 100 μm.

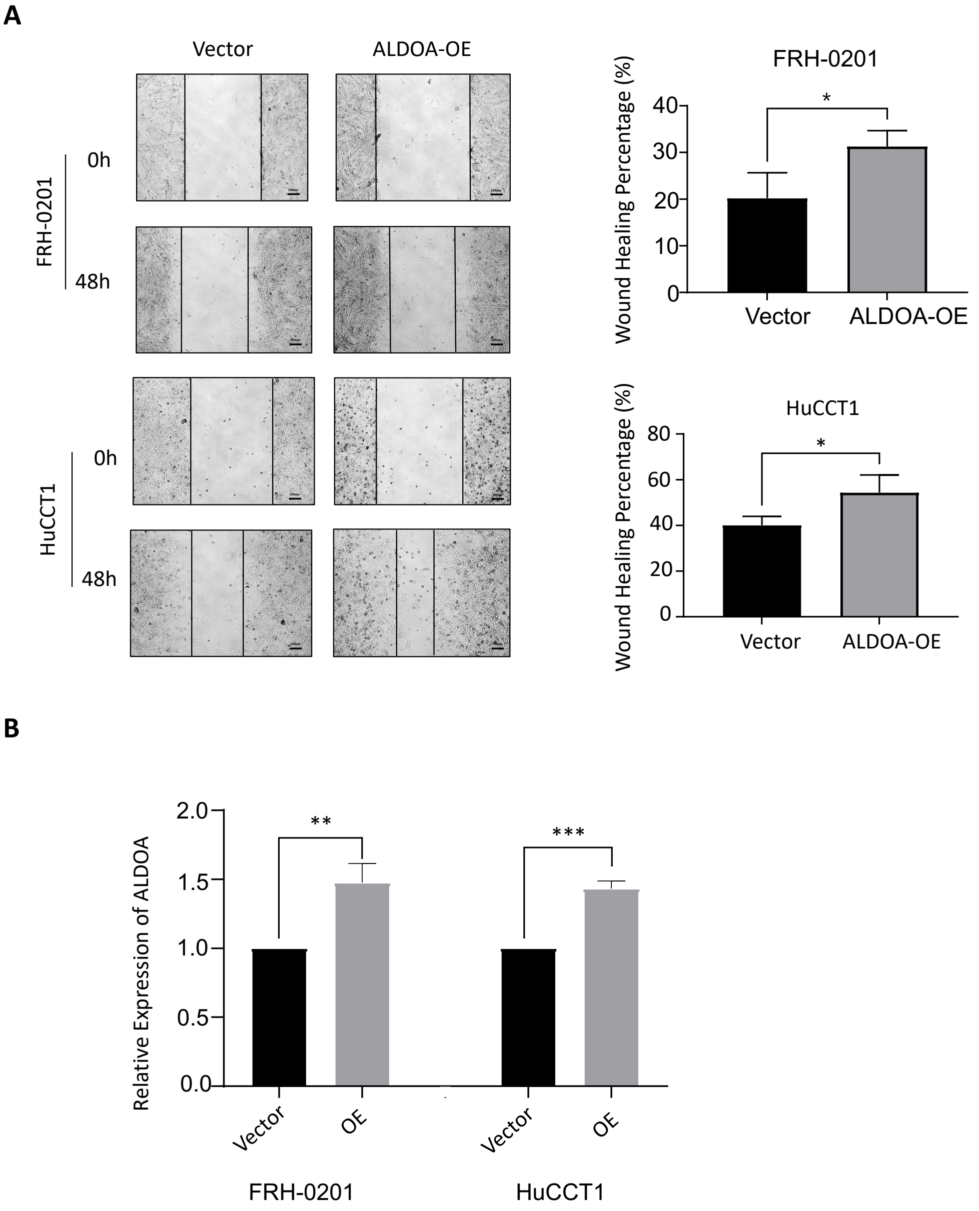

Supplementary Figure 3. A. The level of tumor cell invasion after over expressed of ALDOA by plasmid detected by Wound healing test. B. The Western blot results in Figure 4B were quantified by Image J and analysed by Graphpad. Results are shown as means ± SD. (*, P<0.05; **, P<0.01; ***, P < 0.001). Scale bar, 100 μm.

A

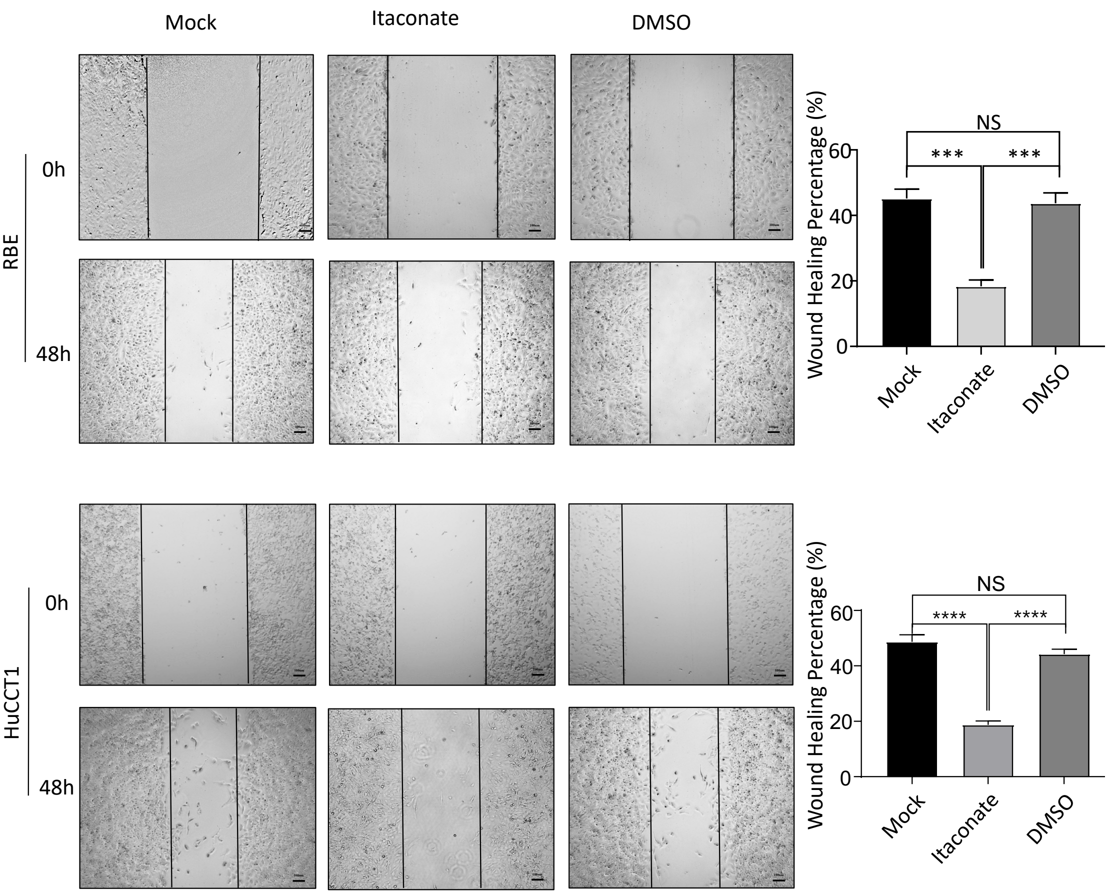

B

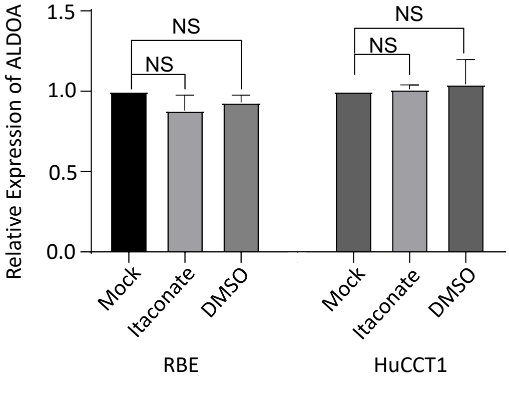

Supplementary Figure 4. A. Wound healing test was used to exam the level of tumor cell invasion after treated by Itaconate or DMSO for 24h. B. The Western blot results in Figure 5B were quantified by Image J and analysed by Graphpad. Results are shown as means ± SD. (***, P < 0.001; ****, P<0.0001). Scale bar, 100 μm.

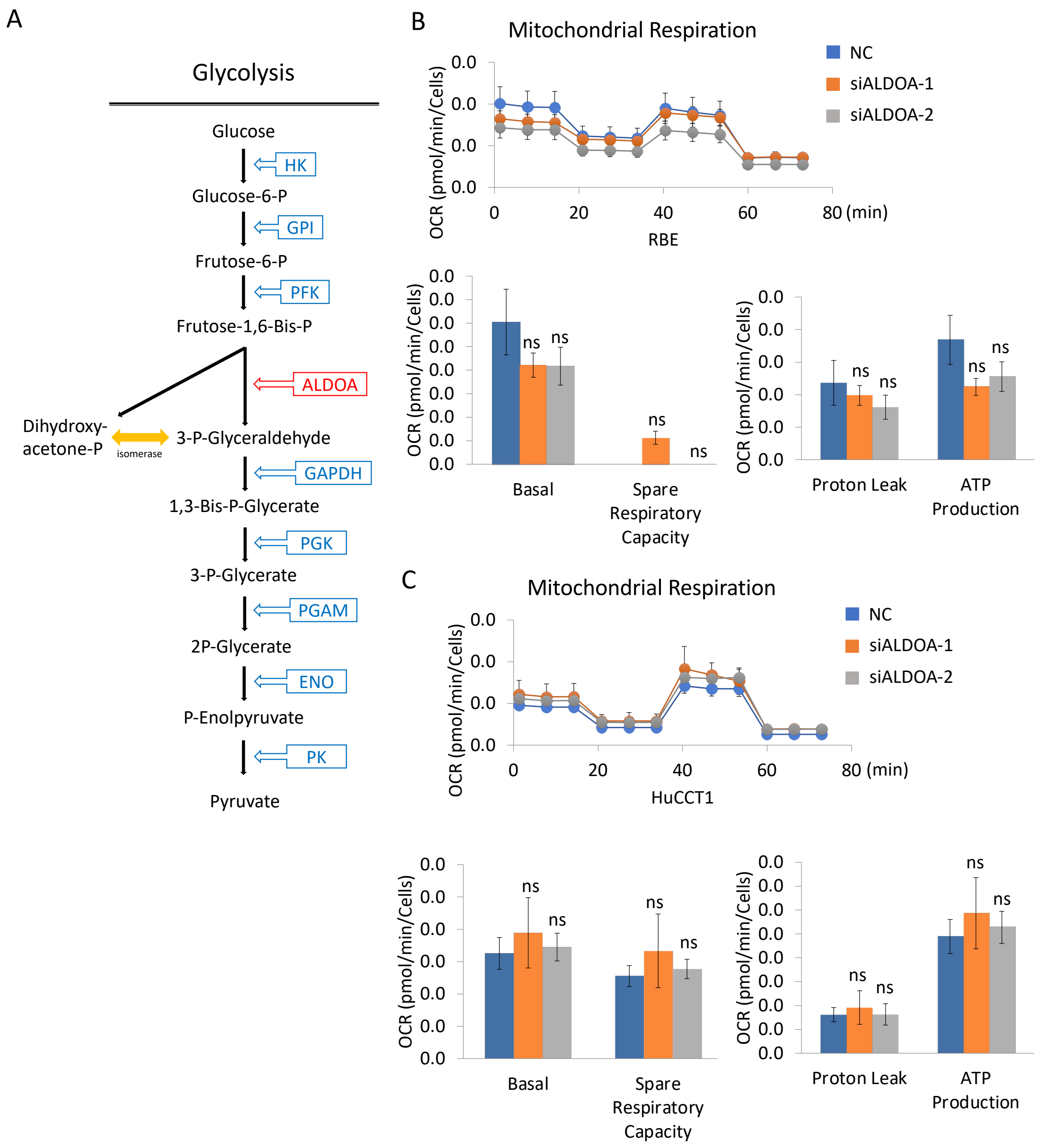

Supplementary Figure 5. A. Flowchart of the enzymes and metabolites that are involved in glycolysis biosynthesis pathway. B. Seahorse was used to detect the Cell Mito Stress level of tumor cells with siRNA knockdown of ALDOA expression in RBE cell line. C. Seahorse was used to detect the Cell Mito Stress level of tumor cells with siRNA knockdown of ALDOA expression in HuCCT1 cell line. Results are shown as means ± SD. (***, $P < 0.001$; ****, $P<0.0001$).
